# The Effect of Circulating Fluidised Bed Bottom Ash Content on the Mechanical Properties and Drying Shrinkage of Cement-Stabilised Soil

**DOI:** 10.3390/ma15010014

**Published:** 2021-12-21

**Authors:** Yuanlong Wang, Yongqi Zhao, Yunshan Han, Min Zhou

**Affiliations:** 1 School of Science, North University of China, Taiyuan 030051, China; zyq18536355860@163.com (Y.Z.); hyswzr@163.com (Y.H.); zhoumin@nuc.edu.cn (M.Z.); 2 Shanxi Graduate Education Innovation Center of Underground Space Engineering, Taiyuan 030051, China

**Keywords:** circulating fluidised bed bottom ash, cement-stabilised soil, optimal ratio, expansibility

## Abstract

This study aimed to determine the effect of circulating fluidised bed bottom ash (CFB-BA) content on the mechanical properties and drying shrinkage of cement-stabilised soil. Experiments were performed to study the changes in unconfined compressive strength and expansibility of cement-stabilised soil with different CFB-BA contents and the underlying mechanisms based on microscopic properties. The results show that CFB-BA can effectively increase the unconfined compressive strength of the specimen and reduce the amount of cement in the soil. When the combined content of CFB-BA and cement in the soil was 30%, the unconfined compressive strength of the specimen with C/CFB = 2 after 60 days of curing was 10.138 MPa, which is 1.4 times that of the pure cement specimen. However, the CFB-BA does not significantly improve the strength of the soil and cannot be added alone as a cementing material to the soil. Additionally, swelling tests showed that the addition of CFB-BA to cement-stabilised soil can significantly reduce the drying shrinkage. This research project provides reference values for the application of CFB-BA in cement–soil mixing piles, including compressive strength and the reduction in the shrinkage deformation of specimens.

## 1. Introduction

In construction, some types of soil pose a greater challenge to the project due to their poor engineering performance. Cement-stabilised soil technology is able to improve the working performance of soil and is often used in building foundation treatment, geological disaster prevention and subgrade reinforcement. Compared with other technologies, cement-stabilised soil has the advantages of low cost, ease of application, and good working performance.

However, increasing cement content does not increase the strength of the soil; thus, other admixtures must be combined with cement-stabilised soil in scenarios where improved strength is required. Admixtures of cement-stabilised soil are mainly divided into three types: cementitious materials, a combination of fibres and cementitious materials, and other materials. The first type, cementitious materials, includes silica fume [1]. However, although only a small amount of silica fume is needed to improve the strength of cement-stabilised soil, its cost is higher than that of cement. Furthermore, adding fly ash [2] and coal-measure metakaolin [3] can improve the late strength of cement-stabilised soil, but this has little effect on the early strength. This is difficult to apply in real-world engineering situations from the perspective of curing time. The second type of admixture involves fibres and cementitious materials [4,5,6]. Adding fibres to cement-stabilised soil can effectively improve its strength. Fibres can enhance the internal structural integrity of the sample and improve the failure mode from brittle failure to plastic failure. The disadvantage of adding fibres to cement-stabilised soil is that if the fibres are not evenly distributed, agglomeration will seriously affect the strength of the specimen. The third type of admixture involves other materials. Adding large quantities of coal gangue to cement-stabilised soil [7] can increase the compressive strength by 80%, but this technology is restricted to specific applications such as roadbed treatment and cannot be widely utilised. Ali R. Estabragh [8] added resin to cement-stabilised soil for unconfined compressive strength testing and found that resin was able to increase the strength of the samples. Fei Xu [9] studied the modification of cement-stabilised soil using polynaphthalene sulfonate (NS) and reported that it can promote the hydration of cement and weaken the adsorption between soil minerals and water molecules, making NS preferentially adsorb soil minerals. The former can delay the formation of AFt (ettringite) and AFm (monosulfate) to compensate for the volume shrinkage of the specimens and increase the strength, and the latter can increase the drying shrinkage of the specimens. At low doses of NS, the volume deformation of the specimens is shown as shrinkage, and volume shrinkage deformation decreases at high doses. Rui Xiao et al. [10] analysed the feasibility of using waste glass aggregate (GA) to replace original aggregate as geopolymer cement through a series of tests and found that GA has potential development value. However, GA reduces the degree of cementation of the specimens, which negatively affects the mechanical properties of the specimen. They also found that higher temperature and humidity can not only increase the strength of the glass powder-based geopolymer, but also reduce the drying shrinkage of the specimen.

CFB-BA is a by-product generated during the sintering process by adding a sulphur-capturing agent (CaO) to the circulating fluidised bed boiler when coal is burned. Circulating fluidised bed technology can reduce sulphur dioxide and other harmful gases produced by burning low-quality coal, but it will produce several times as much waste. There is a need for novel technologies to handle these waste residues. However, due to experimental biases and methodological limitations, it has been difficult to make use of these materials. Nevertheless, progress in this field includes the discovery that CFB ash is a highly absorbent cementitious material, and its mortar has self-hardening and swelling properties, which is attributable to Al_2_O_3_, SiO_2_ and CaO. At present, circulating fluidised bed ash is mainly used in applications such as roadbed treatment [11,12] and new types of cementitious materials and cement mixtures [13,14,15,16,17,18,19]. Currently, the expansibility and pozzolanic activity of CFB ashes can be used to compensate for shrinkage and increase the strength of cement-stabilised soil. However, to avoid detrimental effects on durability, it is necessary to limit the content of SO_3_ [19], add additives [20,21], or implement a pre-hydration treatment [22,23] to reduce hydration-induced expansion.

Despite these advances, there is still little research on mixing CFB-BA in cement-stabilised soil to replace part of the cement and sufficiently improve its strength to achieve industrial waste recycling. Therefore, this project uses loess as the raw material to carry out UCS and expansibility tests on cement-stabilised soil with different contents of CFB-BA, combined with XRD and SEM technology to fully understand the effect of CFB-BA on the mechanical properties of cement-stabilised soil at the different curing ages and to obtain the optimal ratio of CFB-BA and cement.

## 2. Materials and Methods

### 2.1. Material

**Figure 1:**
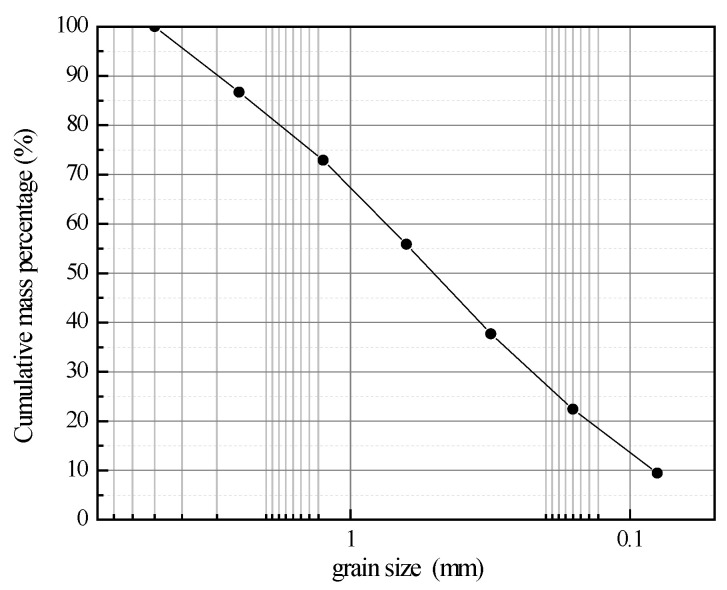
Grading curves.

Soil: The loess from the third terrace in the Jiancaoping District, Taiyuan, Shanxi, was air-dried, crushed, passed through a 5 mm sieve, and subjected to indoor geotechnical tests. The basic physical properties and grading curves are shown in Table 1 and Figure 1, respectively.

**Figure 2:**
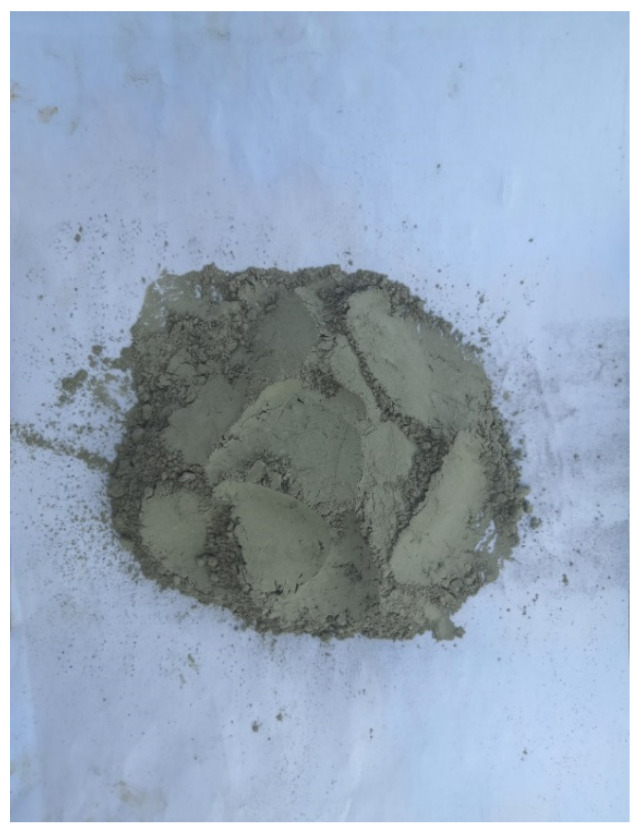
Cement.

Cement: The cement used was P.O42.5 ordinary Portland cement as shown in Figure 2, with a specific gravity of 3120 kg/m^3^ and a specific surface area of 0.342 m^2^/g. The chemical composition of the main substituents is given in Table 2.

**Figure 3:**
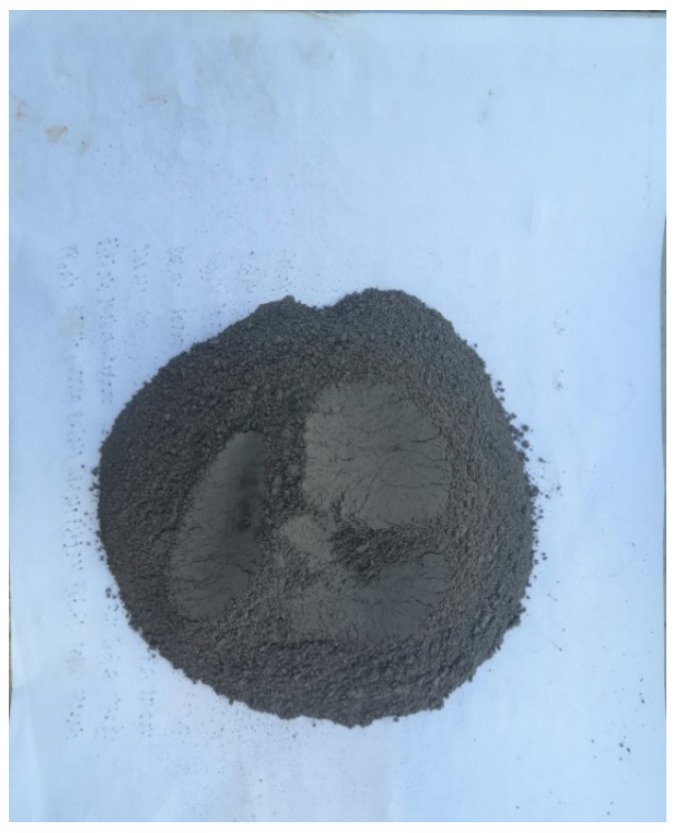
CFB-BA.

CFB-BA: The CFB-BA used herein was produced by Shanxi Pingshuo Coal Gangue Power Plant (which was located in Shuozhou City, Shanxi Province, China) as shown in Figure 3, and its main chemical components are shown in Table 3. CFB-BA is made up of irregularly shaped blocky particles, and the colour of CFB-BA after grinding changes from grey to dark grey. It was found that grinding the CFB-BA could increase its specific surface area and improve the reaction capacity [20,21,22]. Chengzhi [21] determined that the optimal grinding time of CFB-BA was 55 min, with agglomeration occurring after grinding for 72 min. After comprehensive consideration, the CFB-BA used in this study was ground by ball mill for 60 min, and the material retained on an 80 μm sieve was 5.24%.

### 2.2. Sample Preparation and Test Methods

#### 2.2.1. Sample Preparation

Pakbaz M S [24] compared the influence of different preparation methods (dry and wet) on the strength of cement-stabilised soil through unconfined compressive strength (UCS) tests. The strength difference between the two preparation methods became negligible when the curing time was prolonged. Therefore, in order to make the ingredients as uniform as possible, this study adopts a dry method. The mass of cement and CFB-BA were added based on the amount of dry soil, and water was added according to the water-binder ratio (water/(cement + CFB-BA)) = 1.5:1. The specific coordination ratios are shown in Table 4.

Production was carried out according to the proportions in Table 4. Soil, cement, and CFB-BA were poured into containers, then water was added. A hand-held agitator was applied for 5 min to ensure that all components were evenly mixed. The paste produced was placed into a mould with an internal volume of 70.7 mm^3^, then the mould was placed on a vibration table and vibrated for 2–3 min to remove bubbles in the paste. Finally, the surface was scraped and covered with cling film to prevent evaporation of water.

#### 2.2.2. Unconfined Compressive Strength Test

**Figure 4:**
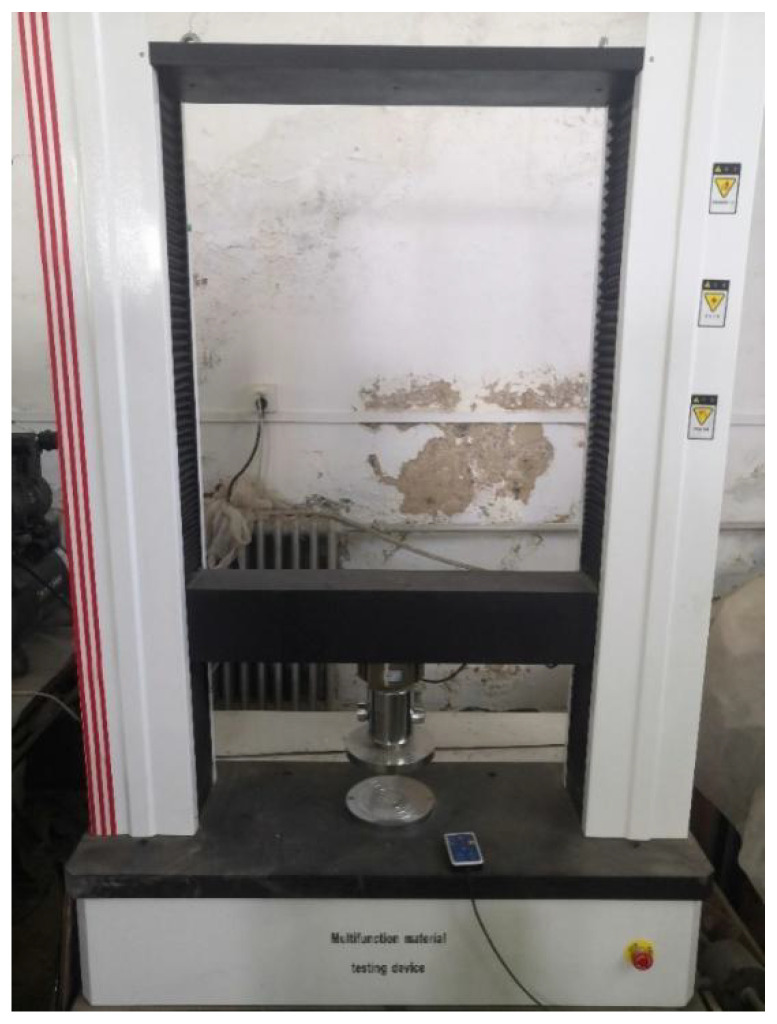
Universal press.

Unconfined compressive strength was tested in specimens with curing times of 7 d, 14 d, 28 d, and 60 d. According to Chinese standard JGJ/T233-2011 (specification for mix proportion design of cement soil), after 36 h, the specimens were demoulded and stored in a curing chamber at 20 ± 2 °C and 95% relative humidity. After curing, specimens were tested for UCS and loaded with a universal press (Figure 4) at a loading rate of 1mm/min.

#### 2.2.3. Stress and Strain Test

The experimental procedures for the elastic modulus of specimens at 28 days all followed the Chinese Standard GB/T50081-2009 (standard for test method of mechanical properties on ordinary concrete). Elastic modulus includes initial secant modulus (E_0_) and secant modulus (E_50_). The secant modulus (E_50_) is defined as the ratio of one half of the compressive strength to the axial strain corresponding to this stress.

#### 2.2.4. Swelling Test

**Figure 5:**
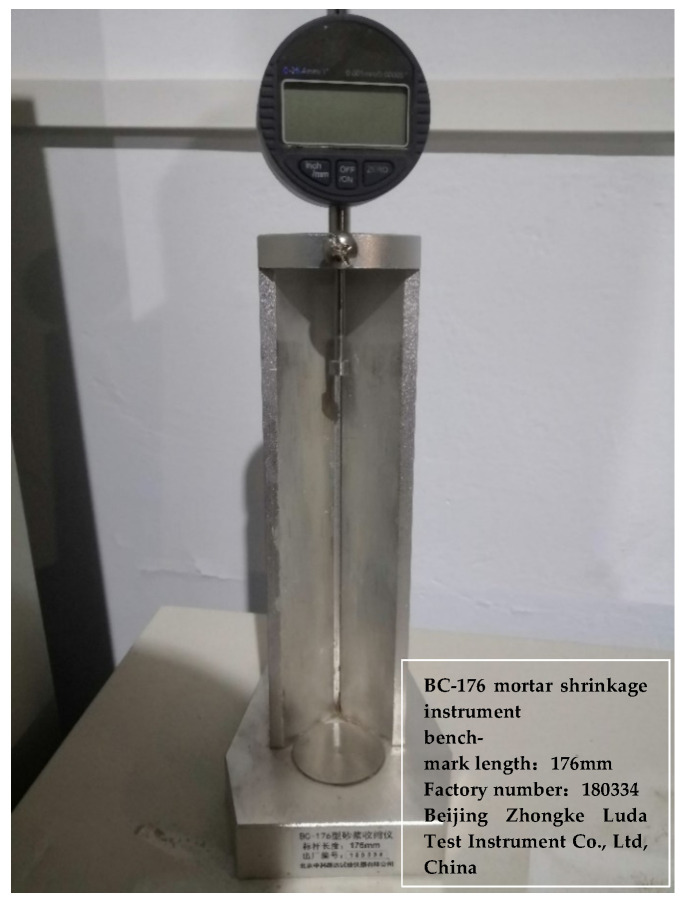
Comparator.

A total of 12 specimens, with total binder contents of 20%, 25%, and 30% (specimen numbers 20-1 to 20-4, 25-1 to 25-5, and 30-1 to 30-3), were selected for the expansion test. The specimen dimensions were 40 mm × 40 mm × 160 mm and the lengths were measured using a comparator (Figure 5). Before demoulding, the specimens and mould were cured together for 1.5 days, and the original lengths of the specimens were recorded. Prior to measurement, specimens were removed from the curing chamber, which had been set at 20 ± 2 °C and 95% relative humidity, and their lengths were recorded after the surfaces had dried. A total of 60 days of test data were collected after demoulding, and then specimens were cured for a further 30 days at the same temperature but without water (humidity ≤ 10%) to determine their expansion rates relative to the standard 60-day curing.

The calculation of the expansion rate for each curing time is as follows:

$$\varepsilon =\frac{L_{t}-L_{0}}{L_{0}}\times 100\%$$

ε—Free expansion rate for a specific age, %
$L_{t}$—The length of the tested specimen at a specific age, mm
$L_{0}$—The initial length of the tested specimen, mm

The length of each specimen was measured three times and the expansion rate was calculated based on the average value.

#### 2.2.5. Microscopic Characteristics of Cement-Stabilised Soil Admixed with CFB-BA

Phase analysis of specimens was conducted using a Japanese Mechanics MiniFlex600 x-ray diffractometer (XRD). The ZEISS MERLIN Compact scanning electron microscope (SEM) was used to observe the internal microstructure and morphology of specimens with different material compositions and to analyse the effect of these changes in microstructure on the macromechanical properties. These two tests were conducted in Taiyuan City, Shanxi Province, China.

## 3. Results

### 3.1. UCS Test

**Figure 6:**
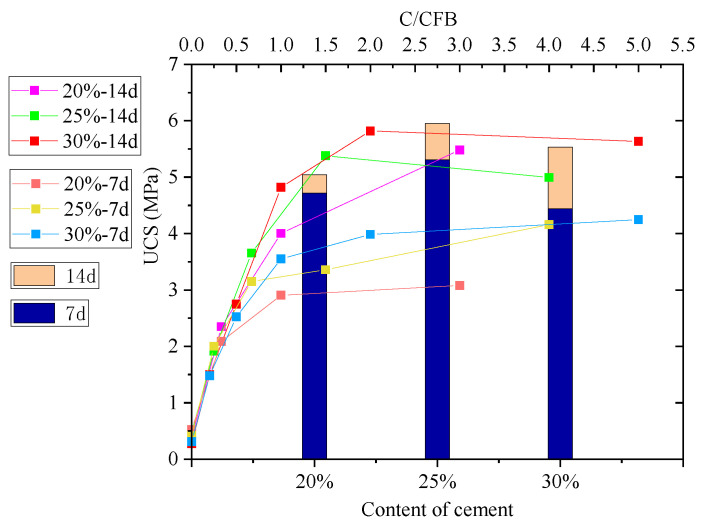
UCS curve of cement-stabilised soil admixed with CFB-BA for 7 days and 14 days (bar graph shows strength of cement-stabilised soil).

**Figure 7:**
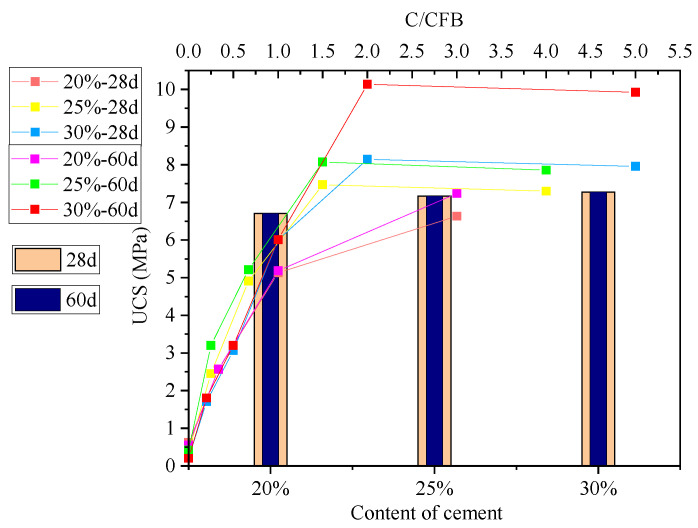
UCS curve of cement-stabilised soil admixed with CFB-BA for 28 days and 60 days (bar graph shows strength of cement-stabilised soil). C/CFB indicates the ratio of cement to CFB-BA.

Figure 6 and Figure 7 show changes in the UCS of cement-stabilised soil admixed with CFB-BA for 7 d, 14 d, 28 d and 60 d, with bar graphs demonstrating the strengths of cement-stabilised soil. After curing for 28 days, the UCS of cement-stabilised soil increased with longer curing times. As C/CFB decreased, the strengths of CFB-BA-cement-stabilised soil specimens with 25% or 30% binder content first increased and then decreased, with the greatest decline at small C/CFB values, indicating that greater amounts of CFB-BA are not necessarily superior. As can be seen in Figure 6, the strength of cement-stabilised soil with 25% cement content was greater than that of specimens with 30% cement content during the first 14 days of curing. This is affected by the comprehensive water content (comprehensive water content = water/(dry soil + binders)). Excessively high comprehensive water contents cause free water to adhere to the surface of the soil particles and hydration products, thereby reducing the bonding area between colloids and soil particles. This results in a decrease in the early strength and an internal pore structure that affects the strength of the product.

As demonstrated in Figure 7, after curing for 28 or 60 days, there is only a small change in the strength of the cement-stabilised soil, which indicates that strength has already developed at 28 days. After 28 days of curing, the UCS of the specimen increased with increasing cement content, but the rate of increase slowed at higher cement contents, indicating that the beneficial effects of cement on the strength of cement-stabilised soil are limited. Therefore, the most economic effect can be obtained at a cement content of 25%. Many factors influence the UCS of the specimen in different ways. Studies show that the optimal amount of cement in soil is 2~25%, which is consistent with these results.

The UCS values of CFB-BA–soil are all lower than 0.5 MPa, which is due to the low degree of cementation in the specimen in the absence of cement. The specimen is also easily damaged during demoulding. Furthermore, because the CFB-BA absorbs water and has poor hydraulic properties, the specimens were in a water-saturated state during the curing period, resulting in low strength. Taken together, these results indicate that cement plays an important role in improving the compressive strength of the CFB-BA soil.

After curing for 7 days, the UCS values of the specimens with CFB-BA was lower than those of cement-stabilised soil, and the strength of the specimens decreased with increasing amounts of CFB-BA replacing cement. Hence, the formation of early strength in these specimens depends mainly on the colloid produced by the hydration of cement, whereas CFB-BA is most important when the curing time exceeds 7 days.

The UCS curve of the CFB-BA–cement-stabilised soil cured for 60 days demonstrates that when the binder content in the soil is equal or greater than 20%, the ratio of cement to CFB-BA cannot be lower than 1.5. When the binder content was 30%, the UCS of the specimen with C/CFB = 2 was 10.138 MPa, which is 1.5 times that of cement-stabilised soil with 20% cement content, and 1.4 times that of the cement-stabilised soil with 30% cement content. Therefore, the optimum amounts of cement and CFB-BA in the soil are 20% and 10%, respectively.

The stress–strain curves were obtained from the UCS tests carried out with a universal press. The stress–strain curve of the specimens with 20% CFB-BA and 10% cement at different curing times was used for the analysis.

**Figure 8:**
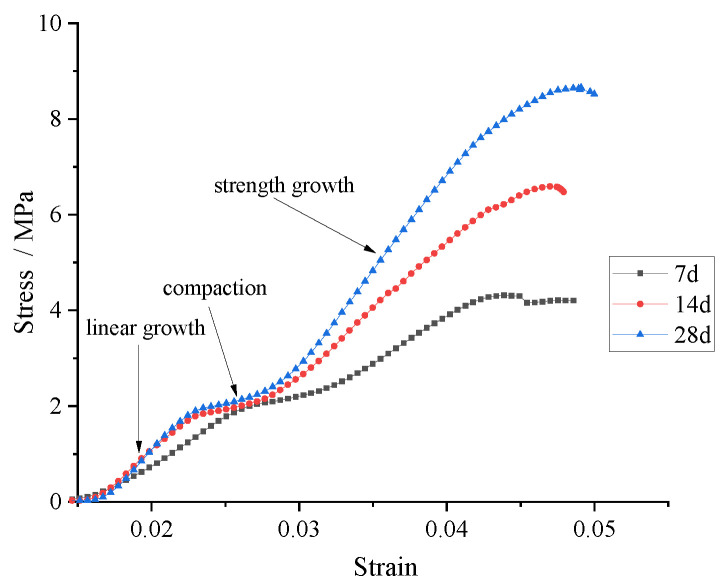
Stress–strain curves of specimens with 20% cement and 10% CFB-BA at different curing times.

From Figure 8, we found that the strength development of CFB-BA-cement–soil specimens can be divided into three stages. The first stage is linear growth, in which the stress–strain relationship is in direct proportion, the slope of the curve is small and the strength development is slow. The second stage is the compaction, in which the strain continues to increase, but the stress increase is small, because the pores are in the process of being compacted. The third stage is the strength growth, in which the stress–strain relationship in the front section also has a linear relationship, and the slope of the curve is slightly larger than that in the first stage, the latter section has a nonlinear relationship and strain continued to increase while the stress increases gradually and eventually reaches the peak strain of the specimen.

### 3.2. Elastic Modulus

**Figure 9:**
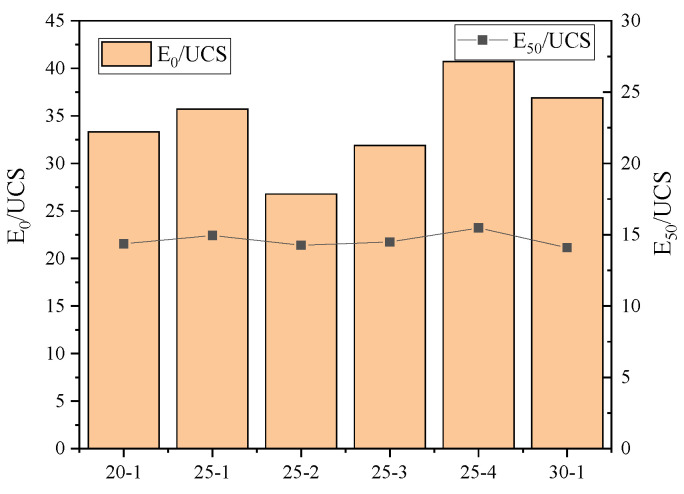
The relationship between the E_0_, E_50_ and the UCS of different specimens (bar graph shows E_0_/UCS).

The relationship between the elastic modulus and compressive strength of the specimens was studied using the stress–strain curve. From Figure 9, we found that the addition of CFB-BA has a greater impact on E_0_/UCS, and the value of E_50_/UCS does not change much regardless of whether there is addition of CFB-BA. When no CFB-BA is added, the value of E_0_/UCS will increase slightly with the increase in cement content. When the amount of cementing material is unchanged, the value of E_0_/UCS will be larger, with increasing amounts of CFB-BA replacing cement. The secant modulus of the specimens E_50_ = (14-15) UCS.

### 3.3. Swelling Test

Previous research has demonstrated that the swelling of CFB-BA admixed with product results in a decrease in the strength [14,16,18,19,21,22,23,25]. Cement-stabilised soil is less compact than concrete, so when an appropriate amount of CFB-BA is mixed into cement-stabilised soil, the swelling of the CFB-BA is conducive to improving its strength. In the current study, although the specimens were vibrated during preparation, there would still be small pores that provide space for the development of crystals.

**Figure 10:**
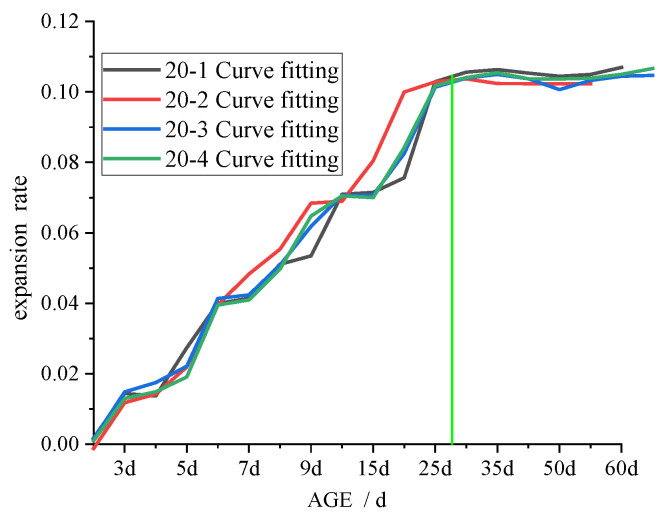
Curve of expansion rate and age of specimen with 20% binder.

**Figure 11:**
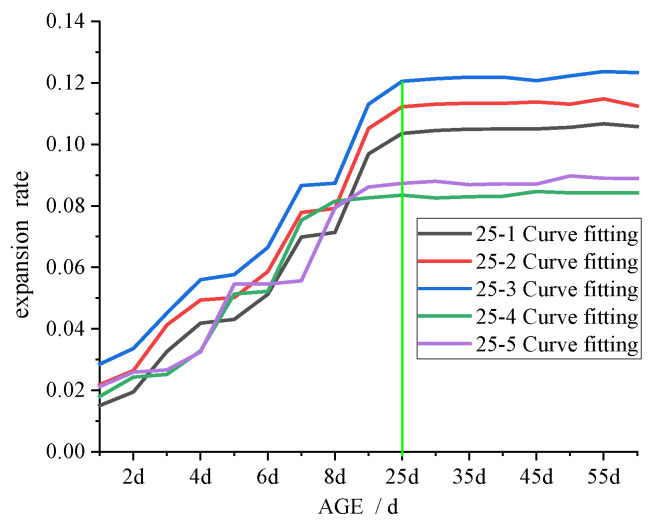
Curve of expansion rate and age of specimen with 25% binder.

**Figure 12:**
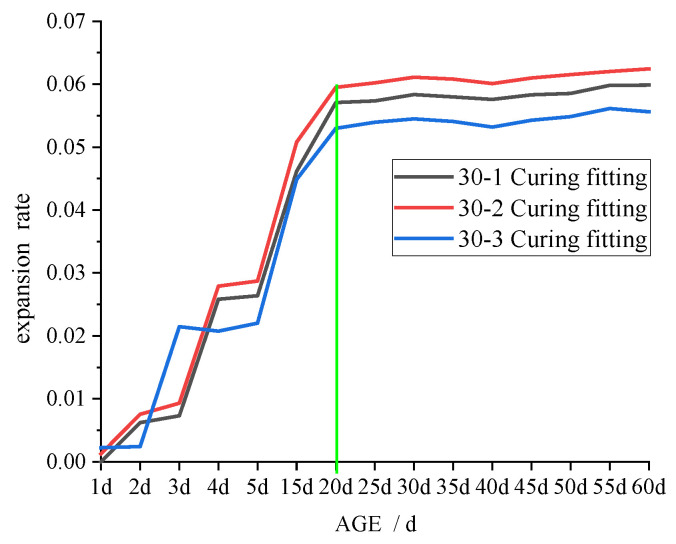
Curve of expansion rate and age of specimen with 30% binder.

The results of the expansion test, showing the relationship between expansion rate and the age of the specimen, is shown in Figure 10, Figure 11 and Figure 12.

As depicted in Figure 10, Figure 11 and Figure 12, increased binder content and comprehensive water content reduced the time required for the specimen swelling rate to stabilise, with 28 days, 25 days, and 20 days required for 20%, 25%, and 30% binder content, respectively. This is attributable to the increased comprehensive water content produced by adding CFB-BA to cement-stabilised soil, which not only improves strength but also minimises the time required for expansion. After curing for 1–2 days, the specimen of CFB-BA–soil became curved due to excessive expansion and the length could not be measured; hence, the expansion test was not carried out.

The hydration products calcium hydroxide (CH) and ettringite (AFt) are the main reasons for the increase in sample volume. In the early stages, the main hydration reactions involve dicalcium silicate (C_2_S), tricalcium silicate (C_3_S), and tricalcium aluminate (C_3_A) in cement and calcium oxide in CFB-BA. The rapid hydration of C_3_A generates a small amount of ettringite in the early stage, which results in the synergistic development of compressive strength and swelling. In the initial stages of CaO hydration, small Ca(OH)_2_ crystals with a size of 10–20 nm are formed. These small crystals later recrystallise to form relatively large crystals [24]. The more free water there is inside the sample, the more conducive it is to the consumption of CaO, which can reduce the pressure of crystal growth produced by Ca(OH)_2_ in the later stage. With the increasing concentration of Ca(OH)_2_, the SiO_2_ and Al_2_O_3_ in the CFB-BA are stimulated to generate C-S-H and C-A-H gels. The dissolution of anhydrite is also accelerated and the formation of ettringite is further promoted. These reactions are expressed in Equations (1)–(3) below:

(1) $$CaO+H_{2}O\to CH$$

(2) $$\begin{array}{l} CH+SiO_{2}+H_{2}O\to CSH\\ CH+Al_{2}O_{3}+H_{2}O\to CAH\\ CaSO_{4}+H_{2}O\to CaSO_{4}•H_{2}O \end{array}$$

(3) $$CAH+CaSO_{4}•H_{2}O+H_{2}O\to AFt$$

As the curing time increases, the strength of the specimen increases, the expansion becomes weaker, and the tensile stress generated by the volume change is less than the tensile strength of the cementitious material. Therefore, the specimen no longer exhibits expansion.

**Figure 13:**
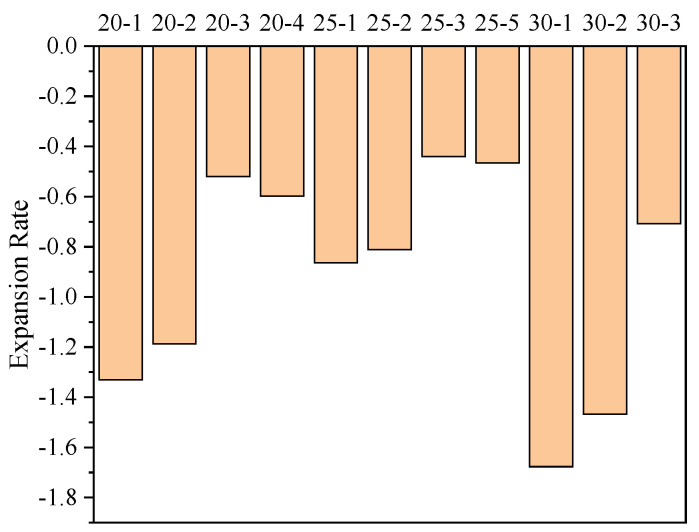
The expansion rates of the specimens cured at a temperature of 20 °C and a humidity of less than 10%.

The expansion rates of specimens cured at the same temperature but in the absence of water are shown in Figure 13.

The shrinkage of cement-based materials is mainly caused by water loss; that is, the free water in the specimen is evaporated or consumed in a reaction. Figure 13 demonstrates that the expansion rate of the specimens with CFB-BA is greater than that of the cement-stabilised soil with identical binder contents, indicating that the addition of CFB-BA in cement-stabilised soil can reduce its drying shrinkage. The addition of CFB-BA reduced the drying shrinkage rates of samples with binder contents of 20%, 25% and 30% by 0.143–0.811%, 0.052–0.86%, and 0.21–0.97%, respectively. The addition of CFB-BA can generate the formation of more rigid crystals such as ettringite in the specimen, which not only fill the pores of the specimen but also form the skeleton to reduce the shrinkage caused by water loss. However, the drying shrinkage only indicates good overall structure, not small porosity.

### 3.4. Composition and Microstructure

**Figure 14:**
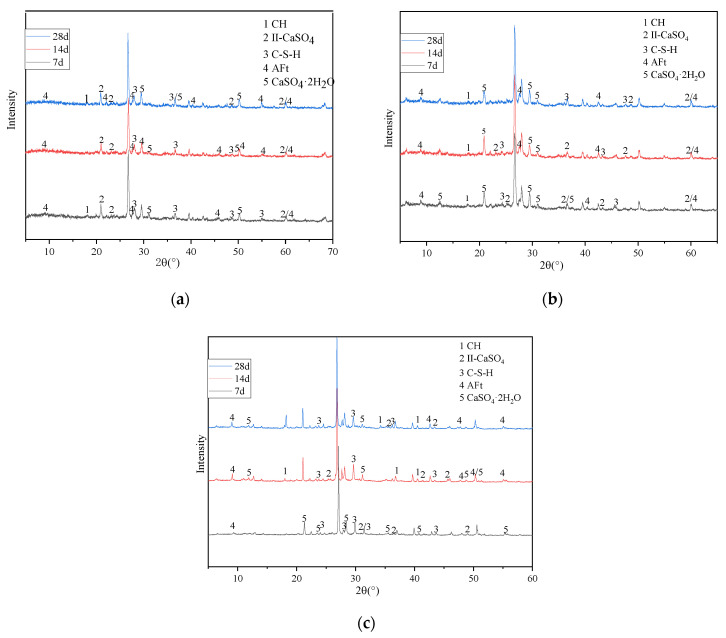
XRD patterns of samples with 30% cementitious material content after curing for 7, 14 and 28 days. (**a**) 30% cement. (**b**) 30% CFB-BA. (**c**) C/CFB = 2.

X-ray diffraction analysis was conducted to determine sample compositions using a Japanese mechanics miniflex 600 X-ray diffractometer (XRD), as shown in Figure 14.

XRD results are greatly influenced by factors such as specimen quality and quantity, as well as instrument properties, but the degree of hydration of the binder can be determined by the diffraction peak intensities of the phase.

Figure 14a shows that the main chemical components of the specimen with 30% cement are II-CaSO_4_, C-S-H, a small amount of AFt and CaSO_4_·H_2_O after curing for 7 days. The main chemical components of the specimen with 30% CFB-BA shown in Figure 14b are II-CaSO_4_, CaSO_4_·H_2_O, AFt and a small amount of C-S-H. In comparison, specimens with 30% CFB-BA produced more gypsum in the early stage. During the process of dissolving anhydrite to gypsum, the solid volume expands 2.26 times; during the formation of ettringite, the solid volume increases 2.22 times, and it is difficult to restrain swelling in the network structure of C-S-H gel. This produces many pores in the specimen, which leads to the loss of effective connection between colloids and failure to produce strength, resulting in a strength of less than 0.5 MPa. The specimen with 30% cement in the early stage can rely on more C-S-H gel to restrain the expansion caused by the hydrolysis of anhydrite and ettringite, filling internal pores and producing early strength. Comparing Figure 14c with Figure 14a, it is evident that adding CFB-BA can increase the content of ettringite and C-S-H gel, the main components of strength, in cement-stabilised soil after curing for 28 days, thus resulting in greater unconfined compressive strength.

**Figure 15:**
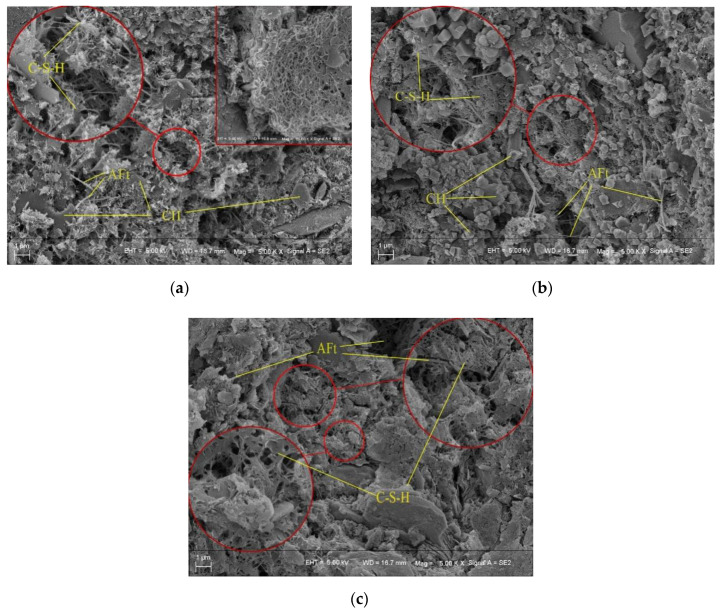
Microscopic images for specimens with 30% binder: (**a**) 30% cement; (**b**) C/CFB = 5; (**c**) C/CFB = 2.

Figure 15a shows that there are large crystals of hexahedral lamellar calcium hydroxide in the specimen, and well-crystallised rod-like and needle-like ettringite crystals in the pores, as well as a spatial network structure composed of flocculated C-S-H gel. Figure 15b displays a large number of well-crystallised small hexagonal calcium hydroxide crystals with a side length of about 1 μm, and many rod-shaped ettringite crystals. Compared with Figure 15a, the addition of 5% CFB-BA in Figure 15b caused an obvious reduction in the pores and a denser structure. Figure 15c shows that a small amount of hexahedral lamellar calcium hydroxide crystals is found in the specimen with C/CFB = 2, and the ettringite crystals are more rod-shaped and overlap with the C-S-H gel to fill in the holes. Most of these remaining holes are smaller than 200 nm, whereas the holes in Figure 15b are larger than 200 nm. Comparing Figure 15a–c, the volume of calcium hydroxide gradually decreased, the ettringite crystals became thicker, and the number and size of pores were reduced. This indicates that the addition of desulphurization slag to cement-stabilised soil promotes the consumption of calcium hydroxide, generates more ettringite, and increases density. Therefore, the UCS values of specimens with C/CFB = 2 were higher than those of C/CFB = 5 when the cementitious content was 30%.

## 4. Discussion

Towards the end of the last century, cement-stabilised soil technology developed gradually and steadily. Today, it is widely used in the field of construction engineering. CFB ash is industrial waste produced as a result of pollution mitigation measures that reduce harmful emissions from the combustion of low-quality coal. At present, there is no fully mature waste treatment method for CFB ash. The expansion of CFB ash affects its ability to be repurposed. Different measures have been proposed to address this issue, such as alternative curing methods, a variety of additives, and a reduction in the quantity used.

Richard J. Deschamp et al. [23] used CFB ash as a filling for roadbed and found that CFB ash continued to show expansion stress two years after construction. Therefore, it was proposed that CFB ash should be pre-hydrated by exposing it to humid air in advance. Guillermo Thenoux et al. [26] studied the potential of CFB fly ash as a soil stabiliser and found that it can improve the strength of soils rich in silica and alumina, but the proportion of CFB fly ash should not be too large due to its expansive properties. Ding T [27] demonstrated that autoclave curing of CFB ash mixed with Portland cement clinker mortar not only improves early strength but also restrains the expansion of CFB ash.

In this project, the maximum content of CFB-BA was 10%. Under standard curing conditions, the strength of soil with 20% cement and 10% ground CFB-BA reached 10.138 MPa after curing for 60 days, which is 1.5 times that of 20% cement-stabilised soil and 1.4 times that of 30% cement-stabilised soil. The actual construction process in this area, the cement content of the cement–soil pile is 25%, and when combined with the high strength and expansibility of cement-stabilised soil mixed with CFB-BA, we believe that this new material can be used in pile foundation engineering. High-strength materials can increase the ultimate bearing capacity of the pile. Additionally, the swelling can improve compaction and increase the friction between the pile and the surrounding soil, which has a positive impact on its bearing capacity. Moreover, it can avoid the brittle failure of the pile due to the restriction of the surrounding soil. When the cement and CFB-BA in the soil are at an appropriate ratio, it can not only improve the working effect of the pile but also reduce the amount of cement and further increase the utilization rate of the CFB-BA in the project.

However, due to the differences between practice and theory, the application of cement-stabilised soil mixed with CFB-BA in pile foundation engineering still needs to be further experimentally verified. The soil quality in different regions is different, so when other soils are used for the above tests, the results may be different. Additionally, adding CFB-BA to cement-soil mixtures reduced the ductility of the specimens. Future work should focus on different soils to carry out the above experiments and carry out related experiments on cement–soil piles mixed with CFB-BA. Although the experimental results are in line with expectations, it is necessary to study the addition of other additives to the CFB-BA-cement-soil to continue to reduce the cement content and improve the brittle failure of the specimens.

## 5. Conclusions

Through UCS tests, swelling tests, and microscopic analysis, this project studied the influence of CFB-BA on the mechanical properties and shrinkage characteristics of cement-stabilised soil and obtained the following conclusions:

- According to the unconfined compressive strength test, the increase in cement content in soil from 25% to 30% resulted in only a small increase in strength, thus the optimum cement content in soil is 25%. Since in the early hydration process of the CFB-BA, more gypsum will be produced, which causes the specimen to expand and cannot effectively improve the strength of the soil, it is not recommended to mix the CFB-BA separately into the soil to increase the unconfined compressive strength.
- At a binder content level of 30%, the UCS of the specimen with C/CFB=2 after curing for 60 days was 10.138 MPa, which is 1.4 times that of the specimen with 30% cement and 0% CFB-BA, indicating that the addition of CFB-BA can reduce the cement content used and increase the compressive strength. When cement and CFB-BA are mixed into the soil, the binder content should not be less than 20% and the ratio of cement to CFB-BA should not be less than 1.5.
- The stress–strain curve of CFB-BA-modified cement-stabilised soil can be divided into a linear growth stage, compaction stage, and strength improvement stage. Regardless of whether CFB-BA is added, the E_50_ and strength of the specimen conform to a certain rule; that is, E_50_ = (14–15) UCS.
- The addition of CFB-BA to cement-stabilised soil can improve the structure of the specimen and reduce shrinkage. The greater the CFB-BA content used, the more obvious the effect, but the compressive strength will also decrease.
- XRD analysis shows that in the early hydration reaction, more CSH gel is more conducive to the improvement of strength. SEM analysis shows that adding CFB-BA to cement-stabilised soil can increase the content of ettringite in the specimens, which is one of the main factors responsible for strength.

## Figures and Tables

**Table 1:** Properties of the soil.

Soil Type	ρg/cm^3^	Natural Moisture Content	PL/%	LL/%	I_P_	Optimum Water Content	ρ_d max_ g/cm^3^	C_u_	C_c_	D_10_	D_50_	D_60_
clayey silt	1.77	5.2%	16.8	27	10.2	13.2%	2.069	9.397	0.868	0.083	0.528	0.78

ρ—specific gravity; PL—plastic limit; LL—liquid limit; I_P_—plastic index; ρ_d max_—maximum dry unit weight; C_u_—coefficient of uniformity; C_c_—coefficient of curvature.

**Table 2:** Chemical composition of ordinary Portland cement.

CaO (%)	SiO_2_ (%)	Al_2_O_3_ (%)	Fe_2_O_3_ (%)	SO_3_ (%)	MgO (%)	K_2_O (%)	Na_2_O (%)	Others
65.19	21.52	4.31	3.38	2.51	2.02	0.61	0.11	0.35

**Table 3:** Chemical composition of CFB-BA.

SiO_2_ (%)	Al_2_O_3_ (%)	CaO (%)	Fe_2_O_3_ (%)	SO_3_ (%)	TiO_2_ (%)	K_2_O (%)
48.41	36.4	6.21	3.42	3.04	0.898	0.674

**Table 4:** Tested mixture ratios of CFB-BA-cement-stabilised soil.

Binders to Soil (%)	Number	Cement to Soil (%)	CFB-BA to Soil (%)
20	20-1	20	0
	20-2	15	5
	20-3	10	10
	20-4	5	15
	20-5	0	20
25	25-1	25	0
	25-2	20	5
	25-3	15	10
	25-4	10	15
	25-5	5	20
	25-6	0	25
30	30-1	30	0
	30-2	25	5
	30-3	20	10
	30-4	15	15
	30-5	10	20
	30-6	5	25
	30-7	0	30

## Data Availability

Data are contained within the article.
